# Cardiovascular Effects of a Novel SIRT1 Activator, SRT2104, in Otherwise Healthy Cigarette Smokers

**DOI:** 10.1161/JAHA.113.000042

**Published:** 2013-06-21

**Authors:** Sowmya Venkatasubramanian, Radzi Mohd Noh, Shruti Daga, Jeremy P. Langrish, Nikhil V. Joshi, Nicholas L. Mills, Ethan Hoffmann, Eric W. Jacobson, George P. Vlasuk, Brian R. Waterhouse, Ninian N. Lang, David E. Newby

**Affiliations:** 1 Centre for Cardiovascular Science, University of Edinburgh, UK (S.V., R.M.N., J.P.L., N.V.J., N.L.M., N.N.L., D.E.N.); 2 GlaxoSmithKline, UK (S.D.); 3 Sirtris, a GSK Company, MA (E.H., E.W.J., G.P.V.); 4 GlaxoSmithKline, PA (B.R.W.)

**Keywords:** cigarette smokers, endothelium, forearm plethysmography, platelet activation, SIRT1, sirtuins, vascular

## Abstract

**Background:**

We examined the effect of the oral SIRT1 activator SRT2104 on cardiovascular function in otherwise healthy cigarette smokers.

**Methods and Results:**

Twenty‐four otherwise healthy cigarette smokers participated in a randomized double‐blind, placebo‐controlled crossover trial and received 28 days of oral SRT2104 (2.0 g/day) or matched placebo. Plasma SRT2104 concentrations, serum lipid profile, plasma fibrinolytic factors, and markers of platelet and monocyte activation were measured at baseline and at the end of each treatment period together with an assessment of forearm blood flow during intra‐arterial bradykinin, acetylcholine, and sodium nitroprusside infusions. Three hours postdose, mean plasma SRT2104 concentration was 1328±748 ng/mL after 28 days of active treatment. Compared with placebo, serum lipid profile improved during SRT2104 administration, with reductions in serum total cholesterol (−11.6±20 versus 6±21 mg/dL), low‐density lipoprotein cholesterol (−10±17 versus 3±21 mg/dL), and triglyceride (−39.8±77 versus 13.3±57 mg/dL) concentrations (*P*<0.05 for all). All vasodilators produced a dose‐dependent increase in blood flow (*P*<0.0001) that was similar during each treatment period (*P*>0.05 for all). No significant differences in fibrinolytic or blood flow parameters were observed between placebo and SRT2014.

**Conclusions:**

SRT2104 appears to be safe and well tolerated and associated with an improved lipid profile without demonstrable differences in vascular or platelet function in otherwise healthy cigarette smokers.

**Clinical Trial Registration:**

http://www.clinicaltrials.gov. Unique identifier: NCT01031108.

## Introduction

Originally identified in yeast, sirtuins represent a class of highly conserved nicotinamide adenine dinucleotide (NAD)–dependent histone deacetylases that have 7 identified members in mammalian species.^1–2^ They have been implicated in the beneficial effects of calorie restriction on longevity in several species and are promising drug targets for a variety of diseases of aging.^3^ Sirtuin (silent mating type information regulation 2 homolog) 1 (SIRT1) is the best‐known member of this class of proteins and is expressed broadly in multiple tissues and highly expressed in the vascular endothelium.^4^ SIRT1 inhibition is associated with vascular dysfunction and arterial thrombosis^5^ as well as alterations in fibrinolysis.^6^ Conversely, SIRT1 activation is associated with improved endothelial function,^7^ enhanced lipid metabolism,^8^ and inhibition of atherogenesis.^9^

Smoking tobacco remains one of the most important and consistent modifiable risk factors for coronary heart disease and is associated with an up to 7‐fold increased risk of nonfatal myocardial infarction.^10^ It is associated with both accelerated atherosclerosis^11^ and a propensity for acute coronary thrombosis.^12–13^ This is mediated through a variety of mechanisms including alterations in vascular, endothelial, fibrinolytic, and platelet function.^14–17^ The precise cellular mechanism for these effects is as yet unknown, but cigarette smoke is associated with oxidative stress, endothelial nitric oxide synthase acetylation, and increased endothelial cell senescence that has been attributed to reduced SIRT1 levels.^4^

To date, there have been few clinical studies to assess the effect of SIRT1 activation in vivo in humans. Therefore, the aim of the present study was to examine the in vivo effects of a novel oral SIRT1 activator, SRT2104, on the lipid profile and vascular, endothelial, and platelet function in otherwise healthy cigarette smokers. We hypothesized that SIRT1 activation could improve the cardiovascular risk profile and reverse or improve the vascular and endothelial dysfunction associated with cigarette smoking.

## Methods

The study was approved by the Research Ethics Committee, was given Clinical Trial Authorization by the Medicines and Healthcare products Regulatory Authority (MHRA), and carried out at the MHRA Phase 1 accredited Wellcome Trust Clinical Research Facility at the Royal Infirmary of Edinburgh, United Kingdom, between June 2010 and September 2011. Written informed consent was obtained from each volunteer, and the study was carried out in accordance with the Declaration of Helsinki.

### Study Participants

Twenty‐four otherwise healthy male and female volunteers aged between 18 and 70 years who smoked ≥10 cigarettes daily for at least 1 year were eligible for the study. Exclusion criteria included the presence of significant comorbidities, chronic illness, renal or liver impairment, history of gastrointestinal diseases or surgeries influencing drug absorption, history of alcoholism, history of neoplastic disease within the last 5 years, a positive urinary test for recreational drugs, pregnancy, and participation in other clinical trials or blood donation within the last 3 months. Eligibility of participants including absence of relevant medical history was confirmed through a standardized form completed by the registered general practitioners after informed consent. Tests for pregnancy (serum human chorionic gonadotrophin [HCG] concentrations at screening and urinary HCG concentrations at study visits) were conducted on all female participants of child‐bearing potential.

### Study Design

**Figure 1.**
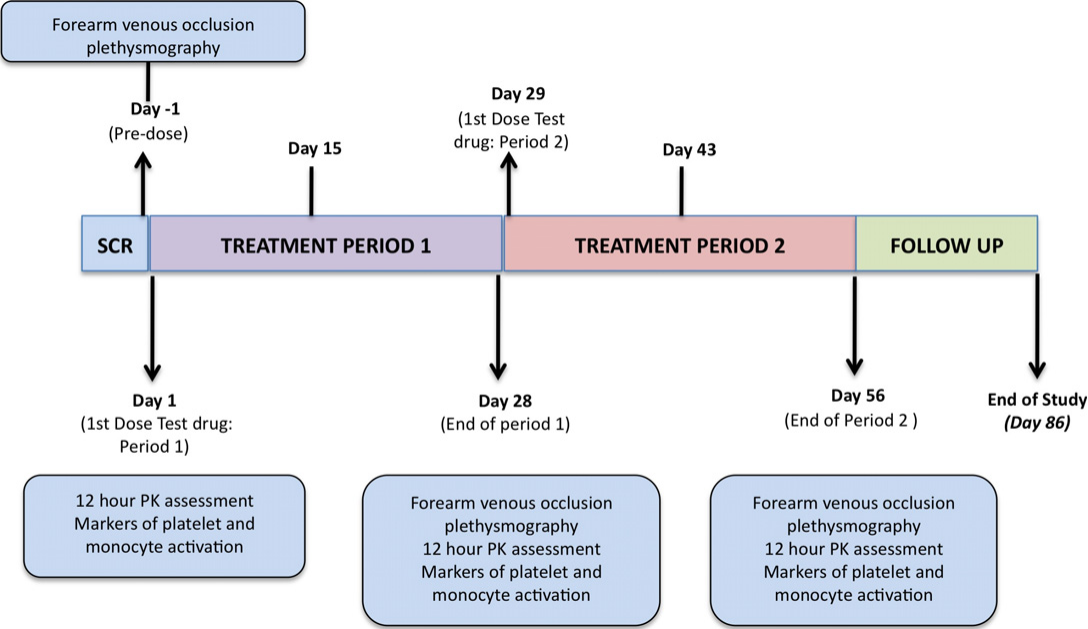
Study design: schematic representation of study design. PK indicates pharmacokinetic; SCR, screening.

This was a prospective double‐blind, randomized, placebo‐controlled crossover study (1:1 SRT2104:placebo). Subjects were randomized to receive 2.0 g daily of oral SRT2104 or matched placebo (Sirtris Pharmaceuticals Inc) for a 28‐day period, followed by crossover to the alternate study arm for another 28 days, giving a total dosing duration of 56 days. An end‐of‐study visit was conducted on day 70, with a phone call follow‐up on day 86. Assessment of drug safety, tolerability, and efficacy on vascular function was carried out at baseline and during and at the end of each treatment period (Figure 1).

### Vascular Studies

Vascular studies were undertaken before and at the end of each 28‐day trial period. All studies were performed with the patient lying supine in a quiet temperature‐controlled (22°C to 25°C) room. Participants were fasted and asked to refrain from smoking for 10 hours before the study and to avoid caffeine and alcohol for 24 hours before the study. Venous cannulas (17G) were inserted into large subcutaneous veins in the antecubital fossae of both arms at the start of the study to facilitate periodic venous sampling. Supine heart rate and blood pressure were monitored at intervals throughout the study using a semiautomated noninvasive oscillometric sphygmomanometer (Omron 705 IT).

### Forearm Venous Occlusion Plethysmography

Forearm blood flow was measured in the infused and noninfused forearms using forearm venous occlusion plethysmography as described previously.^17^ Subjects underwent brachial artery cannulation in the nondominant forearm with a 27 standard‐wire‐gauge steel needle. After a 20‐minute baseline infusion with 0.9% saline, incremental intra‐arterial doses of bradykinin (American Peptide Co) at 100, 300, and 1000 pmol/min (an endothelium‐dependent vasodilator that evokes tissue plasminogen activator [t‐PA] release); acetylcholine (Chem. Pharm Fabrik GmBH) at 5, 10, and 20 μg/min (an endothelium‐dependent vasodilator that does not evoke t‐PA release); and sodium nitroprusside (Hospira Inc) at 2, 4, and 8 μg/min (an endothelium‐independent vasodilator that does not evoke t‐PA release) were infused for 6 minutes at each dose, with a 30‐minute 0.9% saline washout infusion between drugs. The order of drugs was randomized between subjects but kept constant for each subject across the 3 visits.

### Blood Sampling

Paired venous blood samples were obtained from each forearm before and during the infusion of intra‐arterial bradykinin. Samples were collected into acidified buffered citrate (Stabilyte; Trinity Biotech Plc) and citrate (BD Vacutainer; BD UK Ltd) for determination of t‐PA and plasminogen‐activator inhibitor type 1 (PAI‐1) concentrations, respectively. Samples were placed on ice before centrifuging at 2000*g* for 30 minutes at 4°C. Platelet‐free plasma was decanted and stored at −80°C before further analysis. Venous blood samples were collected into EDTA at the beginning and end of the vascular study to determine hematocrit.

Plasma t‐PA antigen and activity (t‐PA Combi Actibind t‐PA ELISA kit; Technoclone, Vienna, Austria) and PAI‐1 antigen and activity (Elitest PAI‐1 Antigen and Zymutest PAI‐1 Activity; Hyphen Biomed) concentrations were determined by enzyme‐linked immunosorbent assays (ELISAs).

### Platelet and Monocyte Activation

Flow‐cytometric measurements of platelet–monocyte aggregation (PMA) and platelet surface expression of P‐selectin and monocyte CD11b expression (Mac‐1/CD11b) were performed at baseline and at the end of each treatment period as described previously.^16,18–19^ Briefly, peripheral venous blood was drawn from a large antecubital vein and anticoagulated with the direct thrombin inhibitor d‐phenylalanine‐l‐arginine chloromethyl ketone (75 μmol/L PPACK; Cambridge Biosciences) and immunolabeled within 5 minutes of phlebotomy for subsequent flow cytometric analysis. Directly conjugated monoclonal antibodies were obtained from DakoCytomation and Serotec. Samples were stained with the following conjugated monoclonal antibodies: phycoerythrin (PE)–conjugated CD14, PE‐conjugated CD62p, PE‐conjugated CD11b, fluorescein isothiocyanate (FITC)–conjugated 42a, and FITC‐conjugated CD14 and appropriate control isotypes. Once stained, samples were incubated for 20 minutes at room temperature before being fixed with FACS‐Lyse (Becton‐Dickinson). All samples were analyzed using a FACS Calibur flow cytometer using CellQuestPro software (Becton‐Dickinson).

Venous blood was collected in citrate at baseline and after each dosing period to assess plasma‐soluble CD40 ligand (sCD40L) concentrations. Blood was centrifuged at 1500*g* for 15 minutes at 4°C, and plasma was decanted and stored at −80°C for further analysis by ELISA (Bender Medsystems).

### Safety and Pharmacokinetic Analyses

Venous blood samples were collected biweekly to measure hematological and biochemical analytes including full blood count, coagulation profile, liver and renal function, creatine phosphokinase, lactate dehydrogenase, lipid profile°C and free fatty acids. Analyses were conducted by the regional clinical hematology and biochemistry reference laboratories using an automated hematology analyzer (XE2100, Sysmex Corporation and ACL TOP, Instrumentation Laboratory), an automated chemistry analyzer using colorimetric, kinetic and enzymatic ultraviolet and color assays (AU2700/AU640 analyzers, Beckman & Coulter), ion‐selective electrodes (sodium, potassium, and chloride assays) and 2‐point and multiple‐point rate assays (Ortho Clinical Vitros 250 analyzer).

Venous blood samples were taken into prelabeled heparinized sodium tubes for pharmacokinetic assessment of plasma SRT2104 concentrations (Simbec Laboratories Limited). Serial blood samples were collected on days 1, 28, and 56 immediately before (0 minutes) and 15, 30, 60, 120, 180, 240, 480, 720 and 1440 minutes following study medication. Plasma was separated by centrifugation of whole blood at 1500*g* at 4°C for 15 minutes, and decanted and stored at −80°C until analyzed.

### Methodology of SRT2104 Analysis

Plasma concentrations of SRT2104 were measured using liquid chromatography with tandem mass spectrometry detection in positive ion mode. High‐pressure liquid chromatography was performed using Betasil silica–100 columns using a Phenomenex C18 guard column.^22^

### Data Analysis and Statistics

Plethysmographic data were analyzed as described previously.^21^ Estimated net release of t‐PA and PAI‐1 antigen and activity was defined as the product of forearm plasma flow (based on blood flow and hematocrit) and the difference in plasma antigen (or activity) concentrations between the 2 forearms. On the basis of previous power calculations,^20^ a sample size of 20 gives 80% power to detect a change in net t‐PA antigen release of 27.0 ng/100 mL of tissue per minute, assuming a standard deviation of 40.0 and a 2‐sided *P*<0.05 (paired *t* test). To account for a 20% dropout rate, we recruited 24 subjects.

Fibrinolysis and forearm blood flow data were analyzed using a linear mixed‐model repeated‐measures analysis of covariance. Treatment differences were investigated in a model adjusting for period, treatment by period, vasodilator dose, treatment by vasodilator dose, and vasodilator dose by period using SAS for UNIX (version 9.1.3 or higher; SAS Institute). Values for these parameters are expressed as model adjusted (least square means) and 95% confidence intervals. Between‐day reproducibility of forearm venous occlusion plethysmography data was assessed using the Bland–Altman method, and coefficient of reproducibility was determined for 95% confidence intervals using the Student *t* distribution. All other values are expressed as mean±SD.

## Results

### Study Participants

Volunteers had a mean age of 38±13 years (median, 37 years) and relatively equal sex distribution (58% male) and were normotensive without any significant coexisting medical conditions. Volunteers had a body mass index of 25±4 kg/m^2^ and a mean cigarette consumption of 17±6 cigarettes per day over 21±14 years. The mean urinary cotinine concentration at screening was 1352±950 ng/mL. All 24 volunteers completed all study visits. Before drug administration, 1 subject was withdrawn from the study because of problems with venous access and was replaced.

### Pharmacokinetics, Tolerability, and Safety

**Figure 2.**
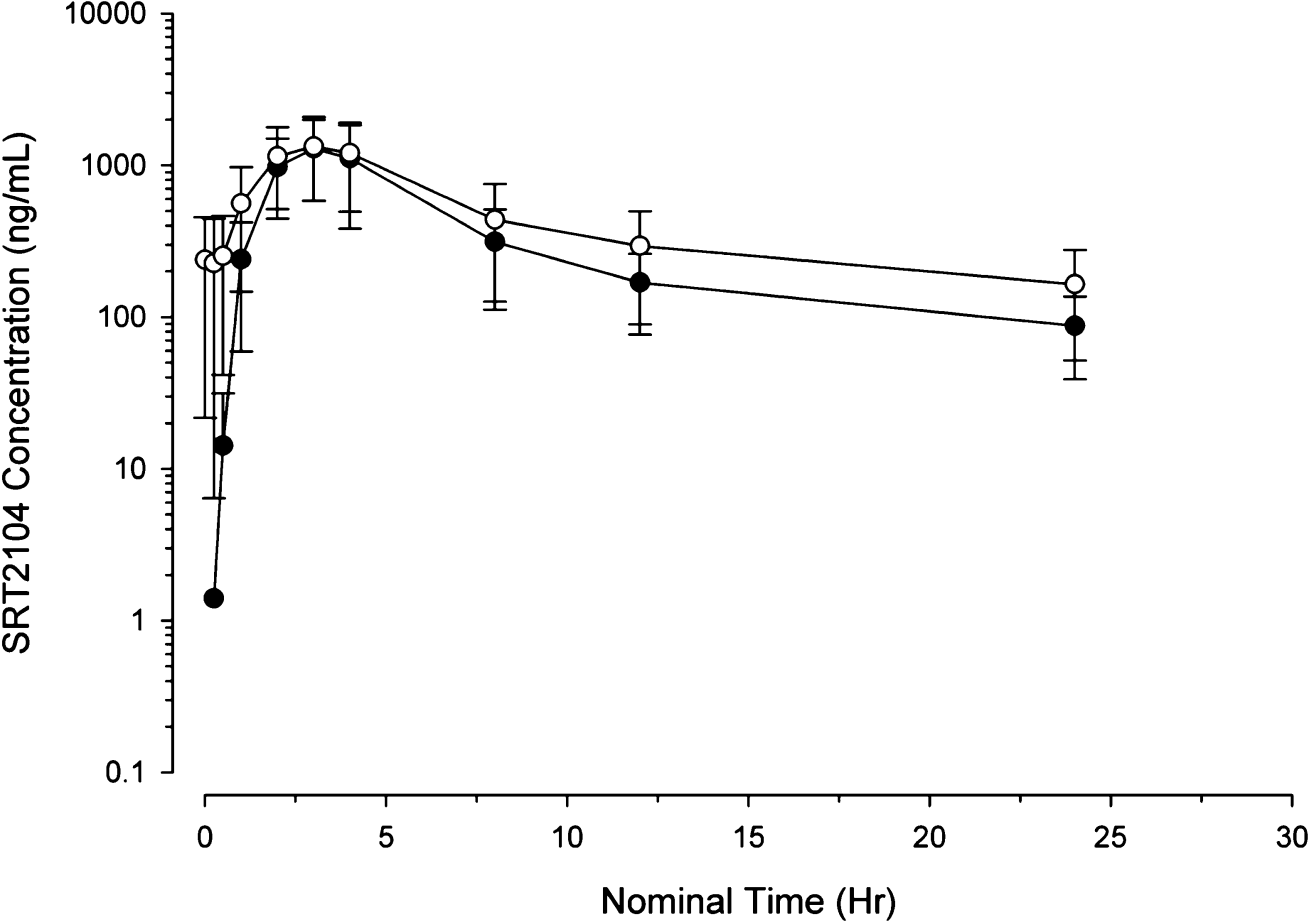
Pharmacokinetics: mean plasma concentration–time curve following oral dosing of SRT2104 on day 1 (closed circles) and day 28 (open circles). Data presented as mean±SD.

Three hours postdose, mean plasma SRT2104 concentration was 1328±748 ng/mL after 28 days of active treatment (Figure 2). The median plasma SRT2104 concentration after 28 days of treatment was 366 ng/mL (IQR, 940 ng/mL). The median time at which the maximum plasma concentration was observed (T_max_) on day 28 of dosing was 3.05 hours, which coincided well with study measurements performed on those days (2 to 4 hours postdose). The geometric mean area under the curve (AUC_0‐τ_) was 6412 h · ng/mL. Consistent with previous observations,^22^ there was substantial intersubject variability in exposure during this study.

All subjects tolerated study medication well. Commonly reported side effects included headache (25%) and rhinitis, nasopharyngitis, and respiratory tract symptoms (17%) (Table 1). The reported adverse events were mild in intensity and resolved without any intervention or sequelae. There were no meaningful differences in the number of events between active treatment and placebo. There was only 1 reported serious adverse event in the study (SRT2104 arm): a traumatic facial bone fracture that was considered unrelated to SRT2104.

**Table 1.** List of Adverse and Serious Adverse Events

System	Symptom (AE/SAE)	Number of Events	
		Placebo (n=24)	SRT2104 (n=24)
Any event	—	18	18
Nervous system disorders	Headache	4	6
	Paresthesia	0	2
	Hypoesthesia	1	2
	Carpal tunnel syndrome	0	1
	Presyncope	1	1
	Burning sensation	0	1
	Sciatica	0	1
Respiratory, thoracic, and mediastinal disorders	Oropharyngeal pain	1	2
	Rhinorrhea	0	1
Gastrointestinal disorders	Flatulence	1	0
	Mouth ulceration	0	1
	Abdominal pain upper	1	0
	Hemorrhoids	1	0
Reproductive system and breast disorders	Dysmenorrhea	3	1
Musculoskeletal and connective tissue disorders	Back pain	2	0
	Musculoskeletal chest pain	1	0
	Musculoskeletal pain	1	0
	Myalgia	0	1
Investigational	Blood bilirubin increased	1	1
	Creatinine phosphokinase increased	1	0
	LDH increased	1	0
	AST increased	1	0
General disorders and administration site conditions	Influenza‐like illness	1	0
	Fatigue	1	0
	Catheter site pain	0	1
	Catheter site rash	1	0
	Catheter site–related reaction	1	0
	Catheter site swelling	1	0
	Edema peripheral	0	1
	Pyrexia	0	1
	Swelling	0	1
Infections and infestations	Nasopharyngitis	0	1
	Rhinitis	2	2
	Upper respiratory tract infection	1	0
	Oral herpes	0	1
	Respiratory tract infection	0	1
Injury, poisoning, and procedural complications	Contusion	1	0
	Excoriation	1	1
	Arthropod bite	1	0
	Facial bones fracture*	0	1
	Laceration	1	0
Skin and subcutaneous tissue	Rash	1	0
Metabolism and nutrition disorders	Decreased appetite	0	1
Vascular disorders	Phlebitis	0	1
Eye disorders	Eye pain	1	0
	Vision blurred	0	1
Ear and labyrinthine disorders	Motion sickness	0	1
Surgical and medical procedures	Vasectomy	1	0

* Serious Adverse Event.

AE indicates adverse event; SAE, serious adverse event; LDH, lactate dehydrogenase; AST, aspartate aminotransferase.

Blood pressure and heart rate remained unchanged throughout the study. There were no effects on cardiac rhythm or the 12‐lead electrocardiogram, and specifically there were no effects on the corrected or uncorrected QT intervals. There were no clinically significant adverse effects involving any of the clinical hematological or biochemical analytes.

### Lipid Profile

Treatment with SRT2104 had a favorable effect on the lipid profile. A statistically significant period effect was observed in the analysis of total and low‐density lipoprotein (LDL) cholesterol concentrations. Baseline values were higher in subjects receiving placebo in the first period. Regardless of treatment arm, the level of change from baseline was greater in period 2 for total and LDL cholesterol and less in period 2 for triglycerides. Adjusted summaries combined over period are presented in Table 2. There was a reduction in total and LDL cholesterol as well as triglyceride concentrations. There was no effect on high‐density lipoprotein concentrations, and the 7% fall in total cholesterol was attributable to the 11% fall in LDL cholesterol concentrations.

**Table 2.** Effect of SRT2104 on Serum Lipid Concentrations

	Placebo (n=22)	SRT2104 (n=20)
Total cholesterol (mean±SD), mg/dL		
Baseline	174±54	176±50
Day 28/56	180±51	164±47
Change from baseline	6±21	−12±20*
HDL cholesterol, mg/dL		
Baseline	46±11	47±10
Day 28/56	50±13	51±16
Change from baseline	3±7	4±7
LDL cholesterol, mg/dL		
Baseline	99±41	99±37
Day 28/56	102±37	88±34
Change from baseline	3±21	−10±17*
Triglycerides, mg/dL		
Baseline	133±110	140±114
Day 28/56	146±149	100±67
Change from baseline	13±57	−40±77*

* *P*<0.05.

HDL indicates high‐density lipoprotein; LDL, low‐density lipoprotein.

### Vasomotor Function

**Figure 3.**
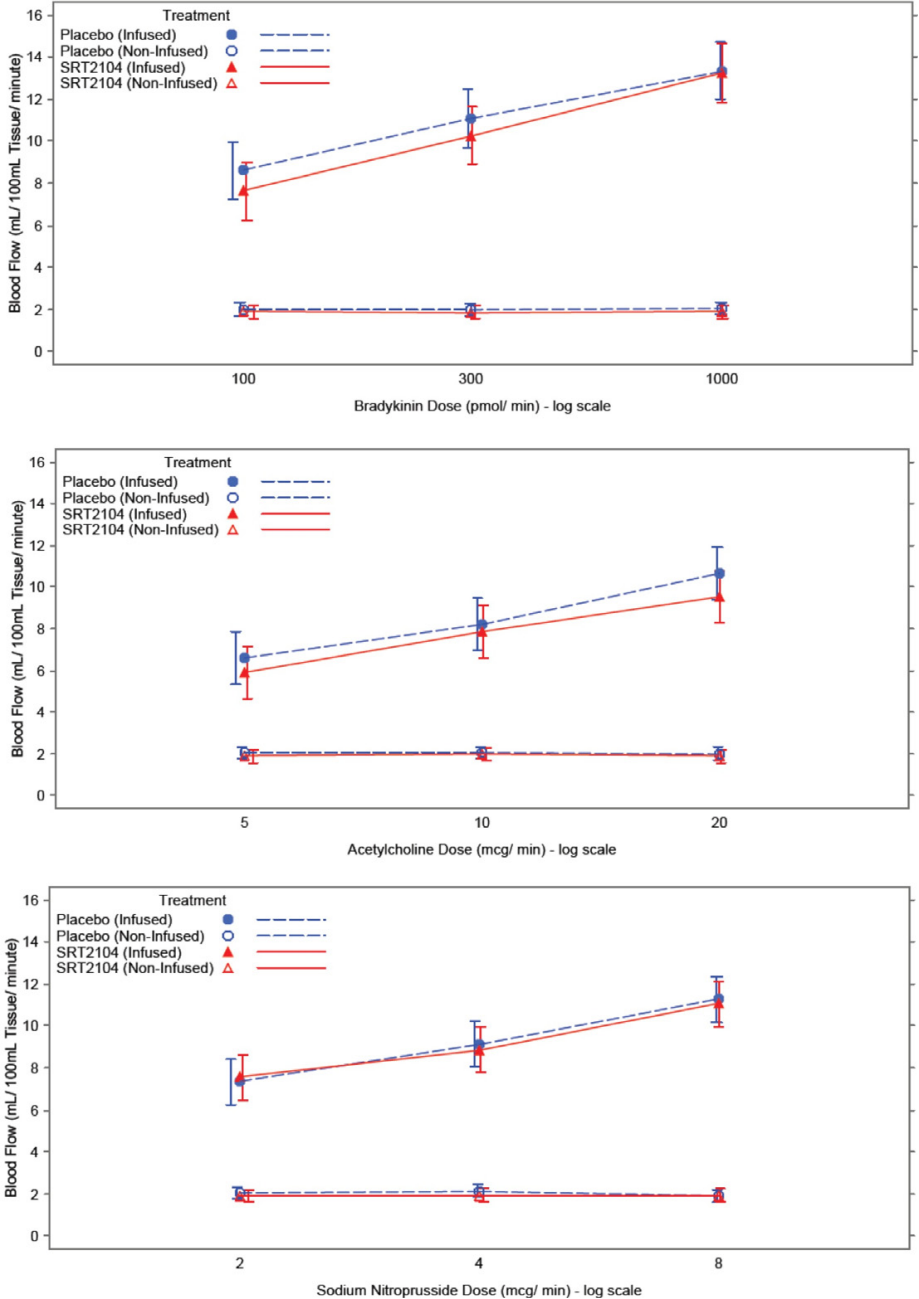
Effect of bradykinin (100, 300, 1000 pmol/min), acetylcholine (5, 10, 20 μg/min), and sodium nitroprusside (2, 4, 8 μg/min) on absolute forearm blood flow. Blue, placebo; red, SRT2104; closed circle, infused forearm blood flow; open circle, noninfused forearm blood flow. Data presented as mean±95% confidence interval.

Noninfused forearm blood flow remained unchanged throughout all assessment periods, as were the predose measurements of blood flow in the infused arm between visits (*P*>0.05). There was a dose‐dependent increase in the infused forearm blood flow with all 3 agonists (acetylcholine, bradykinin, and sodium nitroprusside) in the presence of either SRT2104 or placebo (*P*<0.0001 for all 3 agonists; Figure 3). There were no significant differences in response to either endothelium‐dependent or ‐independent vasodilators in the presence of SRT2104 compared with placebo (bradykinin, *P*=0.1169; acetylcholine, *P*=0.1683; sodium nitroprusside, *P*=0.9039: placebo versus SRT2104). There were no differences in forearm vasodilatation between the baseline and placebo visits of the study for all 3 agonists (*P*=0.5649, *P*=0.4009, and *P*=0.2908 for bradykinin, acetylcholine, and sodium nitroprusside, respectively), confirming the good reproducibility of the measurements (Table 3).

**Table 3.** Between‐Day Repeatability of Forearm Blood Flow

Drug	Dose	Mean of Differences in Forearm Blood Flow (mL/100 mL per minute)	Coefficient of Repeatability (mL/100 mL per minute)
Bradykinin, pmol/min	100	−0.3	5
	300	0.5	6
	1000	−0.2	7
Acetylcholine, μg/min	5	−0.1	8
	10	−0.5	8
	20	0.3	7
Sodium nitroprusside, μg/min	2	0.2	5
	4	0.3	6
	8	0.7	6

Between‐day reproducibility (baseline vs placebo visit) in absolute forearm blood flow for bradykinin (100, 300, 1000 pmol/min), acetylcholine (5, 10, 20 μg/min), and sodium nitroprusside (2, 4, 8 μg/min).

### Endogenous Fibrinolysis and Monocyte and Platelet Activation

There was a dose‐dependent increase in bradykinin‐evoked net t‐PA antigen and activity release (*P*<0.0001 for both) in the infused arm that was unaffected by SRT2104 (*P*=0.3691 and *P*=0.1377, placebo versus SRT2104, for net t‐PA antigen and activity, respectively; Table 4). Plasma plasminogen activator inhibitor‐1 (PAI‐1) activity decreased with time during all study visits (*P*<0.05), consistent with its circadian variation and t‐PA release. Plasma PAI‐1 antigen and activity concentrations were similar in both treatment arms (*P*=0.8877 and *P*=0.6635, placebo versus SRT2104, for plasma PAI antigen and activity, respectively).

**Table 4.** Effect of SRT2104 on Endogenous Fibrinolysis

LS Means+95% CI	Placebo				SRT2104			
	Bradykinin Dose, pmol/min				Bradykinin Dose, pmol/min			
	Saline	100	300	1000	Saline	100	300	1000
Net release t‐PA antigen, ng/100 mL tissue per minute	0.5 (−8.6 to 9.7)	4.7 (−4.4 to 13.9)	8.8 (−0.3 to 18.0)	28.8* (19.6, 37.9)	−1.0 (−10.9 to 9.0)	5.4 (−4.5 to 15.4)	5.6 (−4.7 to 16.0)	38.3** (28.4 to 48.3)
Net release t‐PA activity, ng/100 mL tissue per minute	−0.0 (−4.7 to 4.6)	3.7 (−1.0 to 8.3)	8.6 (3.9 to 13.2)	17.6* (12.9 to 22.2)	0.0 (−5.0 to 5.1)	3.4 (−1.7 to 8.4)	6.5 (1.3 to 11.8)	18.6** (13.5 to 23.6)
Net PAI‐1 antigen, ng/100 mL tissue per minute	−5.7 (−68.2 to 56.8)	—	—	2.9 (−59.6 to 65.5)	−1.7 (−69.7 to 66.3)	—	—	−86.0* (−154.0 to −18.0)
Net PAI‐I activity, LS mean±95% c	−0.0 (−1.2 to 1.2)	—	—	−2.3 (−3.5 to −1.1)	0.0 (−1.3 to 1.4)	—	—	3.9* (−5.2 to −2.6)
Day 28/56								
Net release t‐PA antigen, ng/100 mL tissue per minute	−1.05 (−8.0 to 5.9)	3.9 (−3.1 to 10.8)	8.8 (1.7 to 15.9)	22.4 (15.1 to 29.7)*	−0.1 (−6.7 to 6.6)	2.6 (−4.3 to 9.6)	9.3 (2.3 to 16.2)	30.7** (23.6 to 37.8)
Net release t‐PA activity, ng/100 mL tissue per minute	−0.1 (−3.2 to 3.0)	2.4 (−0.8 to 5.5)	5.8 (2.6 to 9.0)	13.3 (10.1 to 16.6)*	0.0 (−3.0 to 3.0)	2.2 (−0.9 to 5.3)	7.0 (3.8 to 10.1)	18.1** (14.9 to 21.3)
Net PAI‐1 antigen, ng/100 mL tissue per minute	−4.8 (−52.2 to 42.6)	—	—	−20.6 (−70.4 to 29.3)	−0.1 (−45.4 to 45.2)	—	—	−43.7* (−92.4 to 5.1)
Net PAI‐I activity, LS mean±95% c	−0.0 (−1.2 to 1.2)	—	—	−3.6 (−4.9 to −2.4)	0.2 (−1.0 to 1.3)	—	—	−3.3* (−4.5 to −2.1)

Data presented as LS mean±95% confidence interval. t‐PA, tissue plasminogen activator; PAI‐1, plasminogen‐activator inhibitor type 1.

* *P*<0.0001, for dose response to agonist.

* *P*>0.05, SRT2104 vs placebo.

**Figure 4.**
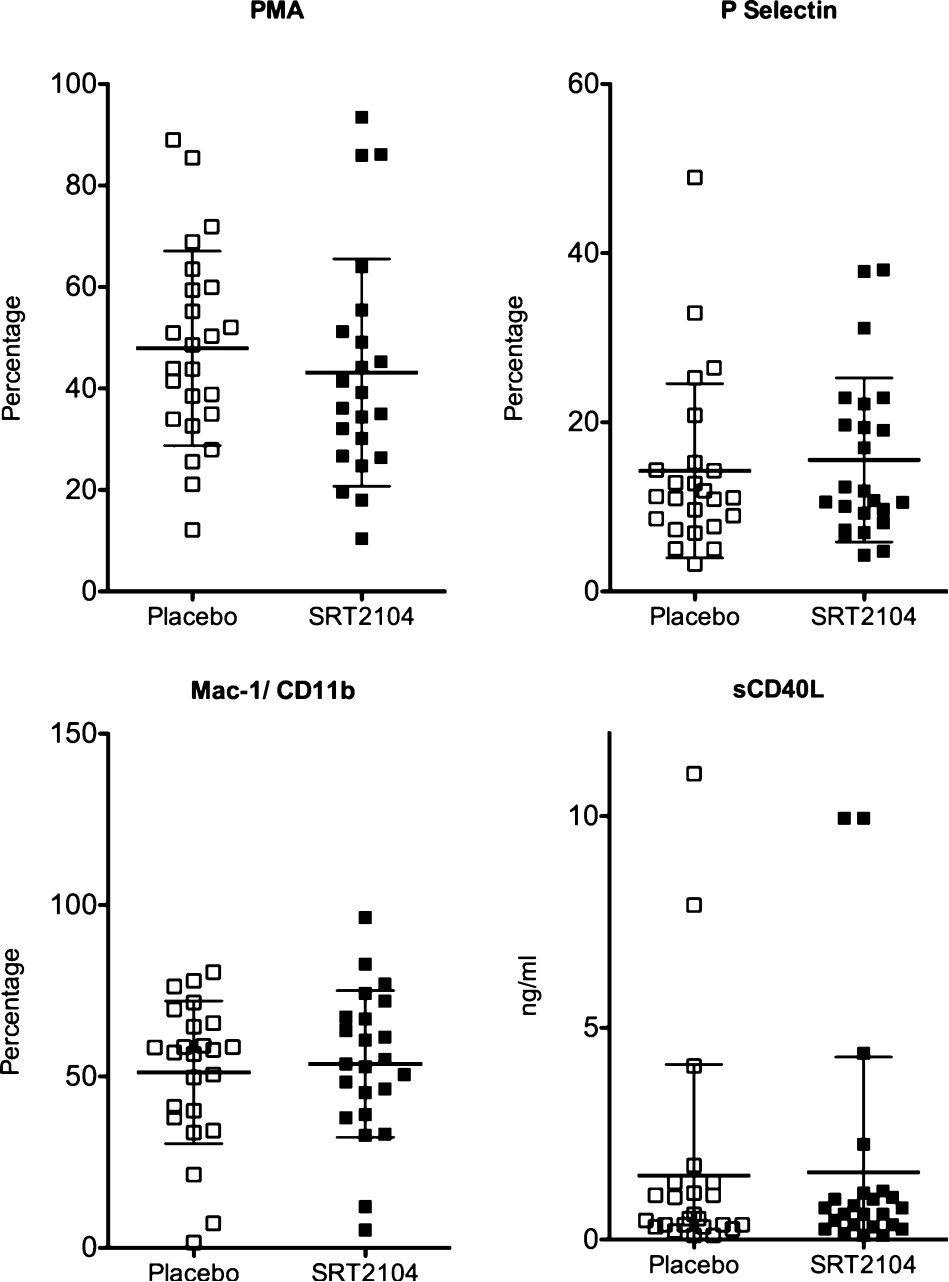
Effect of SRT2104 on markers of platelet and monocyte activation. Data presented as mean±SD. PMA indicates platelet–monocyte aggregate; Mac‐1, macrophage‐1 antigen; sCD40L, soluble CD40 ligand.

SRT2104 had no effect on markers of in vivo platelet or monocyte activation (Figure 4).

## Discussion

In this randomized, double‐blind, placebo‐controlled crossover trial of otherwise healthy cigarette smokers, we have demonstrated that oral SRT2104 is safe and well tolerated at a dose of 2.0 g daily. Importantly, we have shown that treatment with SRT2104 was associated with an 11% mean reduction in serum LDL cholesterol concentrations, but without demonstrable differences in vasomotor function, endothelial function, or platelet activation assessments compared with placebo. The favorable effects on lipid profile suggest that SIRT1 activation may have a beneficial role in patients at risk of developing or with established cardiovascular disease.

Elevated serum cholesterol is an established risk factor for atherosclerosis and coronary heart disease. In general, coronary heart disease risk is reduced by 2% to 3% for each 1% decrease in total cholesterol concentrations.^23^ We observed a 7% mean reduction in serum total cholesterol and an 11% mean reduction in LDL cholesterol concentrations without affecting serum high‐density lipoprotein cholesterol concentrations. The mechanism of this lipid‐lowering effect is not entirely clear but is consistent with observations associated with SIRT1 activation in animals. Resveratrol (3,5,4′‐trihydroxy‐*trans*‐stilbene) is a naturally occurring polyphenolic compound that is believed to confer health benefits through SIRT1 activation.^24^ Resveratrol has been found to lower plasma triglycerides and cholesterol accumulation in guinea pigs^25^ and to suppress atherogenic lesion formation in apolipoprotein E–deficient mice.^26^ Indeed, SRT2104 also lowers triglyceride levels in preclinical murine models of dyslipidemia, diabetes, and obesity as well as improving insulin sensitivity and metabolic function in these animals.^27^ One mechanism whereby SIRT1 activators such as SRT2104 could improve lipid profiles may involve a positive regulatory effect on liver X receptor proteins (LXRs), nuclear receptors involved in cholesterol and lipid homeostasis. Nuclear receptor LXR is a substrate for SIRT1. Li and colleagues have shown that SIRT1 deacetylates and positively regulates this receptor, potentially altering cholesterol transport and metabolism.^28^ Although the exact mechanism of the improved lipid profiles seen with SIRT1 activation remains to be determined, our findings would suggest that SIRT1 activation could provide a therapeutic adjunct to current lipid‐lowering strategies, leading to improvements in cardiovascular disease pathophysiology and thus clinical outcomes.

There are currently no published data directly examining the effects of SIRT1 activation on vasomotor function or endogenous fibrinolysis in vivo in humans. Despite the several beneficial effects of SIRT1 activation on endothelial function observed in preclinical in vitro studies,^6–7,6–31^ we were unable to demonstrate improvements in vascular, endothelial, or platelet function in these otherwise healthy smokers. Why was this?

Did we use appropriate and sufficiently sensitive techniques? Forearm venous occlusion plethysmography is a well‐established technique that has been used extensively over the years to study human vascular physiology and has been considered a gold standard in the assessment of vascular function in health and disease.^32^ Using endothelium‐dependent (bradykinin and acetylcholine) and ‐independent (sodium nitroprusside) vasodilators, we observed a dose‐dependent increase in forearm arterial vasodilatation with all 3 agonists. Our results are comparable with those reported in previously published studies^17,33–35^ in otherwise healthy cigarette smokers including impaired t‐PA release.^15,36–37^ Moreover, our data had low variance and were highly reproducible when we compared the baseline responses with those obtained during placebo administration. Similarly, flow cytometric analysis is considered a sensitive gold standard for measurement of in vivo platelet activation. We have previously shown that in patients with peripheral arterial disease, measurements of platelet–monocyte aggregates are reproducible and consistently reflect other markers of platelet and monocyte activation.^38^ In the present study, we again report comparable levels of platelet–monocyte aggregation^19,39^ that were reproducible between visits.

There is a body of published data that confirms a strong association between cigarette smoking, endothelial dysfunction, and impaired endogenous fibrinolysis.^4,21,17,14,40^ We were interested to see if this vascular and endothelial dysfunction could be improved or reversed by SIRT1 activation. There could be numerous explanations why we failed to achieve improvement with SRT2104 in these parameters in the current study. One possibility is that the SRT2104 exposure achieved in this study did not lead to adequate or consistent SIRT1 activation, which would be required to reverse the vascular and endothelial dysfunction in these smokers. Unfortunately, there is no current biomarker for SIRT1 activation or the ability to measure SIRT1 activation directly in humans. Therefore, we do not have a good understanding of the pharmacokinetic‐pharmacodynamic relationship between SRT2104 drug exposure and SIRT1 activation. Although we were able to demonstrate improved lipid profiles, it is unclear whether the same exposure levels would also lead to improved vascular and endothelial function. There are at least 70 known substrates for SIRT1. SRT2104 may differentially deacetylate certain substrates in preference to others, depending on the precise interaction between SRT2104 and the substrates as well as the level and activity of the substrates in a particular disease state. It is also possible that certain abnormalities may be reversed more readily than others through SIRT1 activation. Although a 28‐day exposure may be adequate for observing improvement in lipid profiles, longer treatment may be required to reverse some of the vascular and endothelial abnormalities. The small sample size of our study may also be a potential limitation. SRT2104 is the first selective SIRT1 activator to be studied in human clinical trials. As the biology of SIRT1 becomes more established and additional data are gathered from small exploratory trials such as this one, the optimal approach for developing SIRT1 activators and identifying disease states with the greatest therapeutic potential will become better defined.

In conclusion, we have demonstrated that the oral SIRT1 activator SRT2104 is safe and well tolerated in otherwise healthy cigarette smokers and provides positive effects on lipid profiles, but were unable to demonstrate beneficial effects on vascular, endothelial, or platelet function compared with placebo.
